# Intra-hippocampal cis-P tau microinjection induces long-term changes in behavior and synaptic plasticity in mice

**DOI:** 10.1186/s12993-023-00211-0

**Published:** 2023-05-25

**Authors:** Bakhtiarzadeh Fatemeh, Shahpasand Koorosh, Shojaei Amir, Fathollahi Yaghoub, Mirnajafi-Zadeh Javad

**Affiliations:** 1 Department of Physiology, Faculty of Medical Sciences, Tarbiat Modares University, 14115-331, Tehran, 1411713116 Iran; 2 Department of Brain and Cognitive Sciences, Cell Science Research Center, Royan Institute for Stem Cell Biology and Technology, ACECR, Tehran, Iran; 3 Institute for Brain Sciences and Cognition, Tarbiat Modares University, Tehran, Iran

**Keywords:** Alzheimer’s like disease, Cis P-tau, Dorsal and ventral hippocampus, Learning and memory and synaptic plasticity

## Abstract

**Background:**

Alzheimer's disease is accompanied by an abnormal high accumulation of cis-P tau. However, the long-term changes in behavior following tau accumulation remains under debate. The present study investigated the long-term effects of tauopathy on learning and memory, synaptic plasticity, and hippocampal cell numbers.

**Results:**

Cis-P tau was microinjected into the dorsal hippocampus to generate Alzheimer’s like-disease model in C57BL/6 mice. Cis-P tau injected animals showed a significant impairment in learning and memory in Y-maze and Barnes maze tests. In another group of animals, the generation of long-term potentiation (LTP) was evaluated in hippocampal slices 7 months after cis-P tau injection. LTP induction was disrupted only in the dorsal but not ventral hippocampal slices. The basal synaptic transmission was also reduced in dorsal hippocampal slices. In addition, hippocampal sampling was done, and the number of cells was assessed by Nissl staining. Obtained results indicated that the number of survived cells was significantly reduced in the dorsal and ventral hippocampus of cis P-tau injected animals compared to the animals in control group. However, the decrement of cell number was higher in the dorsal compared to the ventral hippocampus.

**Conclusions:**

In conclusion, intra-hippocampal cis-P tau injection produced learning and memory impairment at 7 months after its injection. This impairment might result from LTP disruption and a significant decrease in the number of neurons in the dorsal hippocampus.

**Graphical Abstract:**

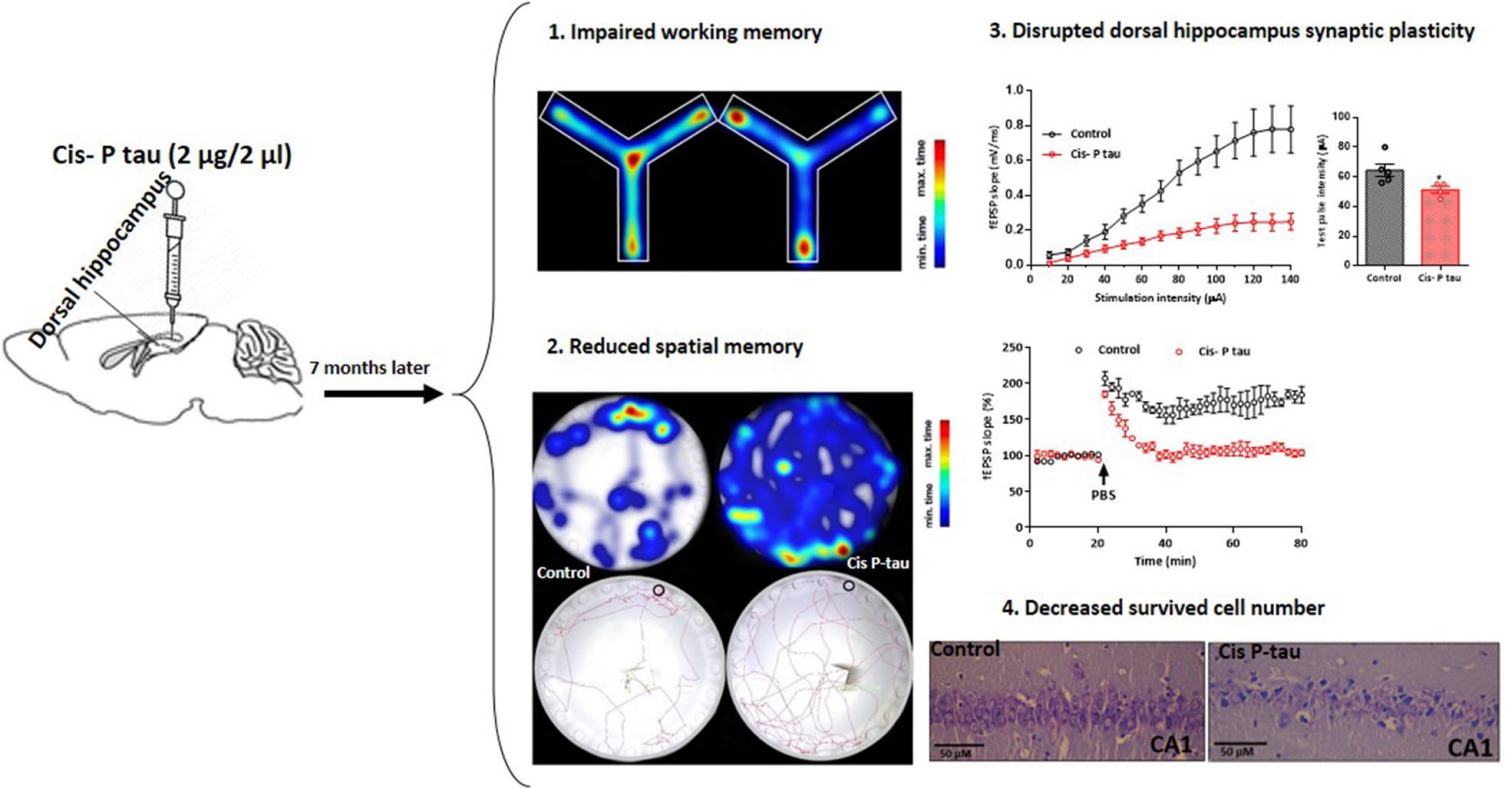

## Background

Alzheimer's disease (AD) is an age-related, progressive, and irretrievable neurodegenerative disorder. The clinical manifestation of AD is a progressive loss of cognitive ability and daily function activities [1, 2]. The most common pathophysiology of AD is an unusual extracellular accumulation of amyloid-β peptide (Aβ) as amyloid and senile plaques and hyperphosphorylated tau protein aggregated as intracellular neurofibrillary tangles (NFTs) [3]. Tau protein is known as a microtubule-associated protein that is mainly expressed in the brain. The main functions of tau are stabilizing and coordinating the molecule's movement along the microtubule, which is strongly regulated by phosphorylation. Phosphorylated tau has two isoforms: trans P-tau is physiological, promoting microtubule assembly, whereas the cis form is pathogenic [4].

The mechanisms of phosphorylation and hyperphosphorylation of tau protein are unknown. Hyperphosphorylated tau, especially cis P-tau, aggregates in some neurodegenerative diseases named tauopathies [4–8]. Tauopathies are progressive neurodegenerative disorders pathologically determined by tau deposits in the brain, such as Alzheimer's disease, frontotemporal dementia, and chronic traumatic encephalopathy [9, 10].

Several pathophysiological events occur after cis P-tau accumulation in the neurons, for example, microtubule networks and axonal mitochondrial transport disruption, propagation of cis P-tau to other neurons [11], activation of several kinases [12, 13], and finally, cell apoptosis. Cis P-tau is aggregated more than trans P-tau because it is resistant to dephosphorylation and degradation, cannot reinforce microtubule assembly, and is more prone to aggregation [4]. Twenty hours after the onset of tauopathy, cis P-tau was dramatically aggregated in the cerebral cortex and remained high for up to 2 months, and was propagated from the cerebral cortex into the hippocampus 6 months later [11]. Therefore, the accumulation of cis P-tau and the possibility of dementia and AD is raised to 30 years later [14].

Pourhamzeh et al. [15] confirmed that bilateral intra-hippocampal injection of cis P-tau could produce Alzheimer-like disease in rats. They reported that cis P-tau increased β-amyloid accumulation and tau protein aggregation in the hippocampus and neocortex and impaired learning and memory in the Morris water maze at 2, 4, and 8 weeks after injection. In line with this data, another study showed that P-tau accumulation following traumatic brain injury results in Alzheimer-like changes and impairment in spatial learning and memory in the Morris water maze 6 weeks after P-tau accumulation. However, these changes disappeared at 7 months after cis-P tau injection [16].

**Fig. 1:**
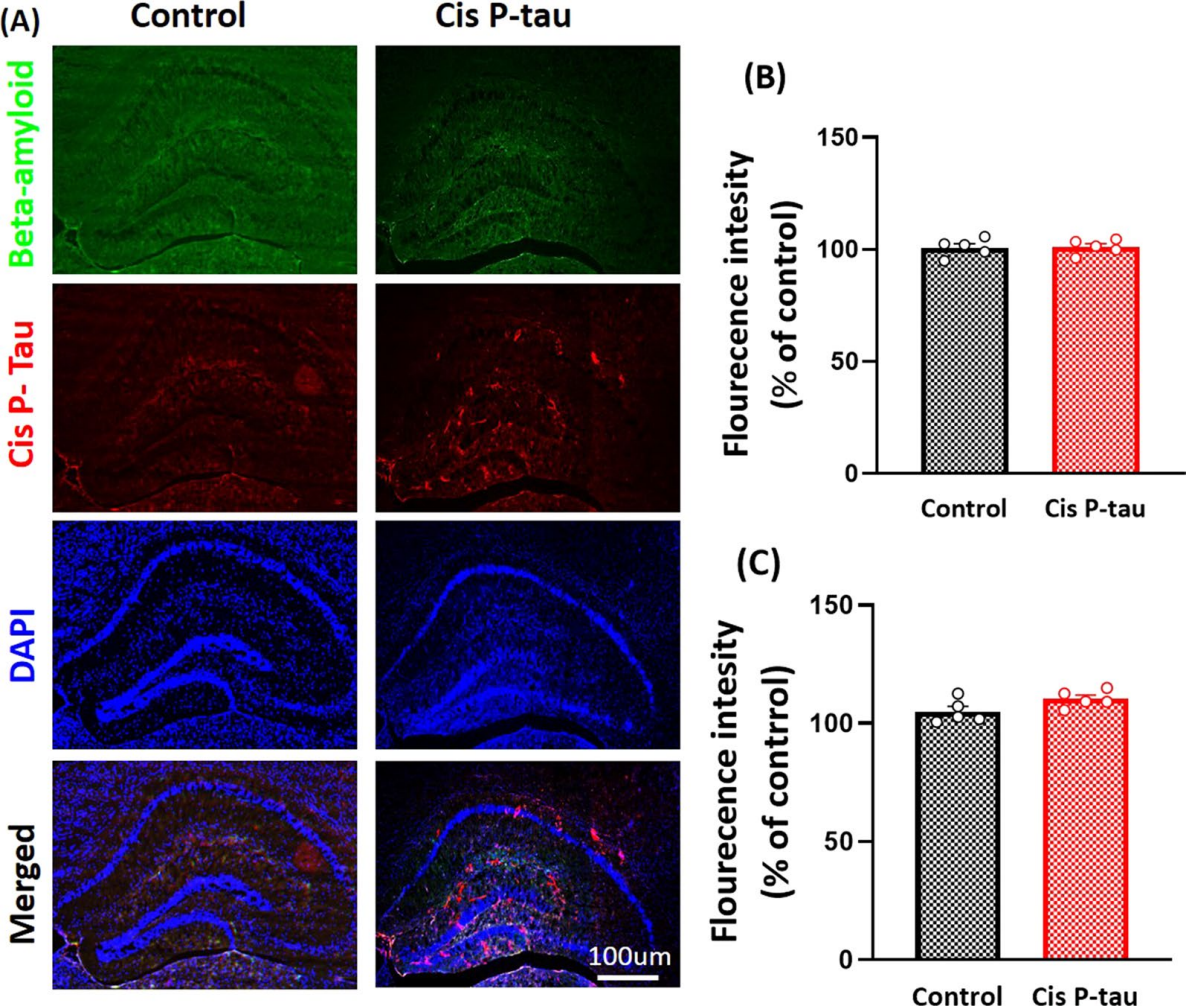
Cis P-tau and β-amyloid accumulation decreased at 7 months after intra-hippocampal cis Pinjection. **A** Cis P-tau and amyloid beta staining is shown in hippocampal area in control and cis P-tau groups. Quantification of β-amyloid **B** and cis P-tau **C** immunostaining showed that there was no hippocampal accumulation at 7 months after cis P-tau injection. All data show mean ± SEM

In addition to memory impairment, pathological tau aggregation in the synapses can promote synaptic loss and synaptic plasticity impairment [17]. The density of NFTs is significantly correlated with synaptic loss and cognitive reduction, indicating that the pathological tau may be a pathogenic factor in AD [18]. Therefore, given that pathologic tau protein disappears in the brain after 7 months, in this study, we want to know whether dorsal hippocampal injection of cis P-tau causes impairment in learning and memory and disruption in synaptic plasticity in dorsal and ventral hippocampus in 7 months after the injection (Fig. 1).

## Results

### Cis P-tau and β-amyloid accumulation disappeared at 7 months after intra-hippocampal cis P-tau injection

At the first step, β-amyloid and cis P-tau accumulation was assessed in the dorsal hippocampus by immunostaining. Previous experiment showed a significant increase in β-amyloid and cis P-tau accumulation up to 2 months after intra-hippocampal cis P-tau injection [15]. Seven months after intra-hippocampal cis p-tau injection, no β-amyloid accumulation was observed and there was no statistical significant between cis P-tau and control groups (*P* = 0.9118). However, there was a slight cis P-tau accumulation in the cis P-tau group, although it was not significant compared to the control group (110.3 ± 1.613% vs. 105 ± 2.22% respectively; *P* = 0.0895).

### Intra-hippocampal cis-P tau resulted in working memory impairment

**Fig. 2:**
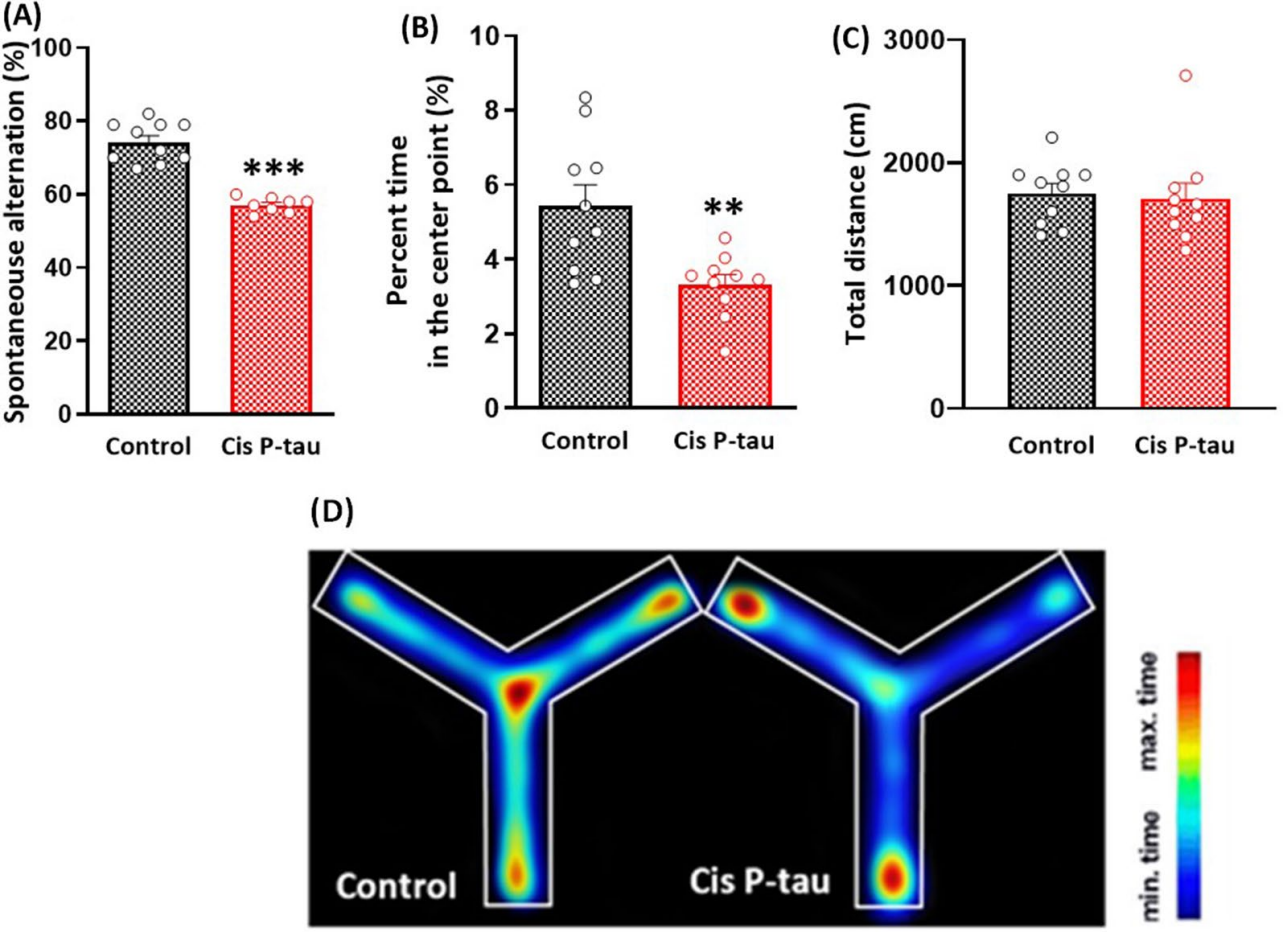
Impaired working memory in mice at 7 months after intra-hippocampal cis P-tau injection. The percentage of spontaneous alternation (**A**) and the percentage of time spent in center point (**B)** significantly decreased at 7 months after intra-hippocampal cis P-tau injection in cis P-tau compared to control group. There was no significant difference in total distance between cis P-tau and control groups (**C)**. Color maps depict the mean spontaneous alternation across all animals in each group, showing the percent time in each arm during the whole eight-min test duration (**D**). All data represents mean ± SEM. **P < 0.01 and *** P < 0.001 compared to the control group

Working memory was evaluated by the Y maze test at 7 month after bilateral intra-hippocampal injection of cis-P tau. Unpaired t-test showed an impaired percentage of spontaneous alternation in the cis-P tau group compared to the control group (57.13 ± 0.72 vs. 74.30 ± 1.73 respectively; *P* < 0.001; Fig. 2A). The percent time spent in center point decreased in cis P-tau compared to control group (3.32 ± 0.27 vs. 5.4 ± 0.57 respectively, *P* < 0.001; Fig. 2B). The total distance did not affect by intra-hippocampal cis P-tau injection (Fig. 2C). These results indicated that cis- P tau administration might cause working memory impairment at 7 month after injection.

### Cis P-tau led to spatial learning and memory damage

**Fig. 3:**
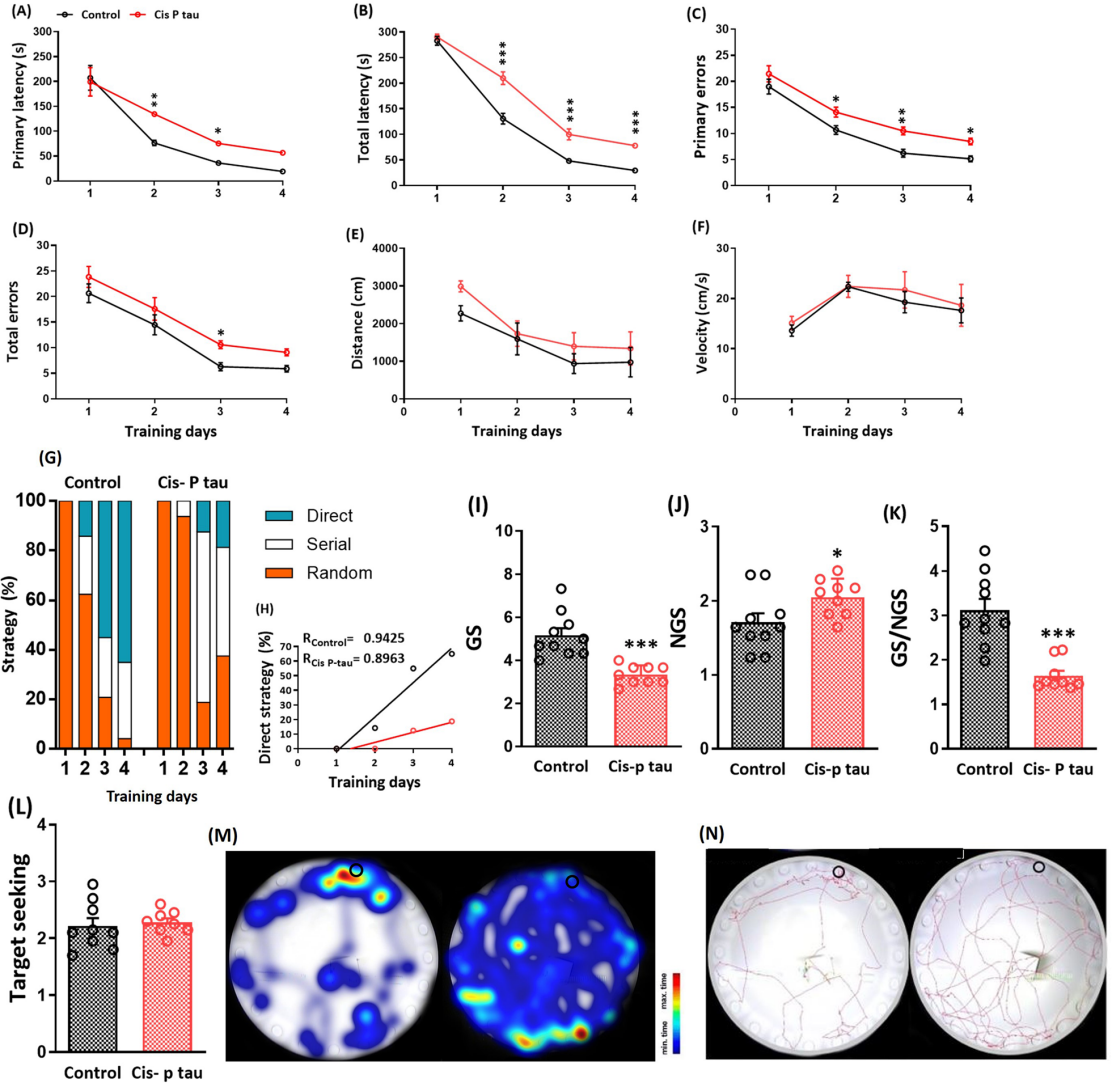
Impaired learning and spatial memory in mice 7 months after intra-hippocampal cis P-tau injection. Graphs shows the effect of intra-hippocampal cis P-tau injection on primary latency **A** total latency **B** primary errors **C** total errors **D** traveled distance **E** velocity **F** strategy **G** and hole exploration frequency in the goal sector (GS) **I** non-goal sector (NGS) **J** GS/NGS ratio **K** and target-seeking activity **L** in the Barnes maze test at 7 months after cis P-tau injection compared to the control group. All data represent mean ± SEM. * P < 0.05, ** P < 0.01, and *** P < 0.001. Color maps **M** shows the percent time in each location of the maze on the probe day in control and cis P-tau groups. A sample of exploration path of animals in control and cis P-tau groups on the probe day is also showing **N**. The black circle marks the location of the escape box in **M** and **N**

Spatial learning and memory were assessed at 7 months after bilateral intra-hippocampal injection of cis-P tau in the Barnes maze test. A two-way ANOVA followed by Sidak's multiple comparisons test showed a higher primary latency to find the goal box in the cis P-tau compared to the control group on day 2 (134.67 ± 2.59 vs. 76.64 ± 5.25; *P* < 0.01), day 3 (75.55 ± 4.12 vs. 36.42 ± 1.97; *P* < 0.05) and day 4 (56.796 ± 2.017 vs. 19.304 ± 2.021; *P* < 0.05) (Fig. 3A). Similarly, the total latency to find the goal box increased in the cis P-tau group compared to control group on day 2 (209.80 ± 12.27 vs. 130.66 ± 10.59; *P* < 0.001), day 3 (99.80 ± 10.85 vs. 48.07 ± 4.01; *P* < 0.001) and day 4 (77.83 ± 4.34 vs. 29.43 ± 4.63; *P* < 0.001) (Fig. 3B).

Primary and total errors were evaluated by two-way repeated measures ANOVA and Sidak's multiple comparisons post hoc test. The primary error was significantly increased in cis P-tau compared to the control group on day 2 (14.11 ± 0.96 vs. 10.69 ± 0.83; *P* < 0.05), day 3 (10.51 ± 0.74 vs. 6.2 ± 0.77; *P* < 0.01), and day 4 (8.48 ± 0.64 vs. 5.15 ± 0.56; *P* < 0.05) (Fig. 3C). The total error was significantly higher in the cis P-tau group compared to the control group only on day 3 (10.58 ± 0.75 vs. 6.29 ± 0.77; *P* < 0.05) (Fig. 3D). There was no significant difference in total distance and velocity in the cis P-tau compared to the control group. These data showed that cis P-tau injection did not interfere with the animal's locomotors activity (Fig. 3E, F).

The strategy to find the goal box was also assessed in these two groups. The random strategy shows the deficiency in animal’s learning and memory. Therefore, animals search the entire maze environment to find the goal box. In serial strategy, the animal's learning increases, and the subject can find the goal box using its memory. Finally, the animal detects the exact location of the goal box using peripheral cues and memory in direct strategy. All (100%) animals in both groups used a random strategy due to a lack of familiarity with the environment on day 1. During the training days, subjects in the control group used serial and direct strategy on day 2 (23.33% and 14.17% respectively), day 3 (24.15% and 55.01% respectively), and day 4 (30.82% and 65.01% respectively). (Fig. 3G, H). However, animals in the cis P-tau group did not learn to use direct strategy and mostly used random and serial strategies on days 2 (93.75% and 6.25% respectively), day 3 (18.75% and 67.85% respectively), and day 4 (37.5% and 43.75% respectively) (Fig. 3G, H). The high percentage of random and serial strategies throughout the training demonstrated that the cis P-tau group could not learn the goal box's location and could not memorize it.

Goal sector (GS) exploration significantly decreased in the cis P-tau group compared to control (3.33 ± 0.15 vs. 5.17 ± 0.33; *P* < 0.001). In addition, non-goal sector (NGS) exploration was significantly increased in the cis P-tau group compared to control (2.05 ± 0.08 vs. 1.20 ± 0.10; *P* < 0.05) during the probe test (Fig. 3I, J). GS/NGS ratio as a GS preference or a spatial memory index was significantly decreased in the cis P-tau group compared to the control (1.65 ± 0.11 vs. 3.13 ± 0.25; *P* < 0.001, Fig. 3K). Target-seeking was also calculated in these two groups, and there was not any significant difference between cis P-tau and control groups (Fig. 3L). These data showed that animals in both groups explored all of the holes equally; however, the subjects in cis P tau group could not memorize the location of the target hole and goal box.

### Cis P-tau decreased basic synaptic transmission on dorsal hippocampus

**Fig. 4:**
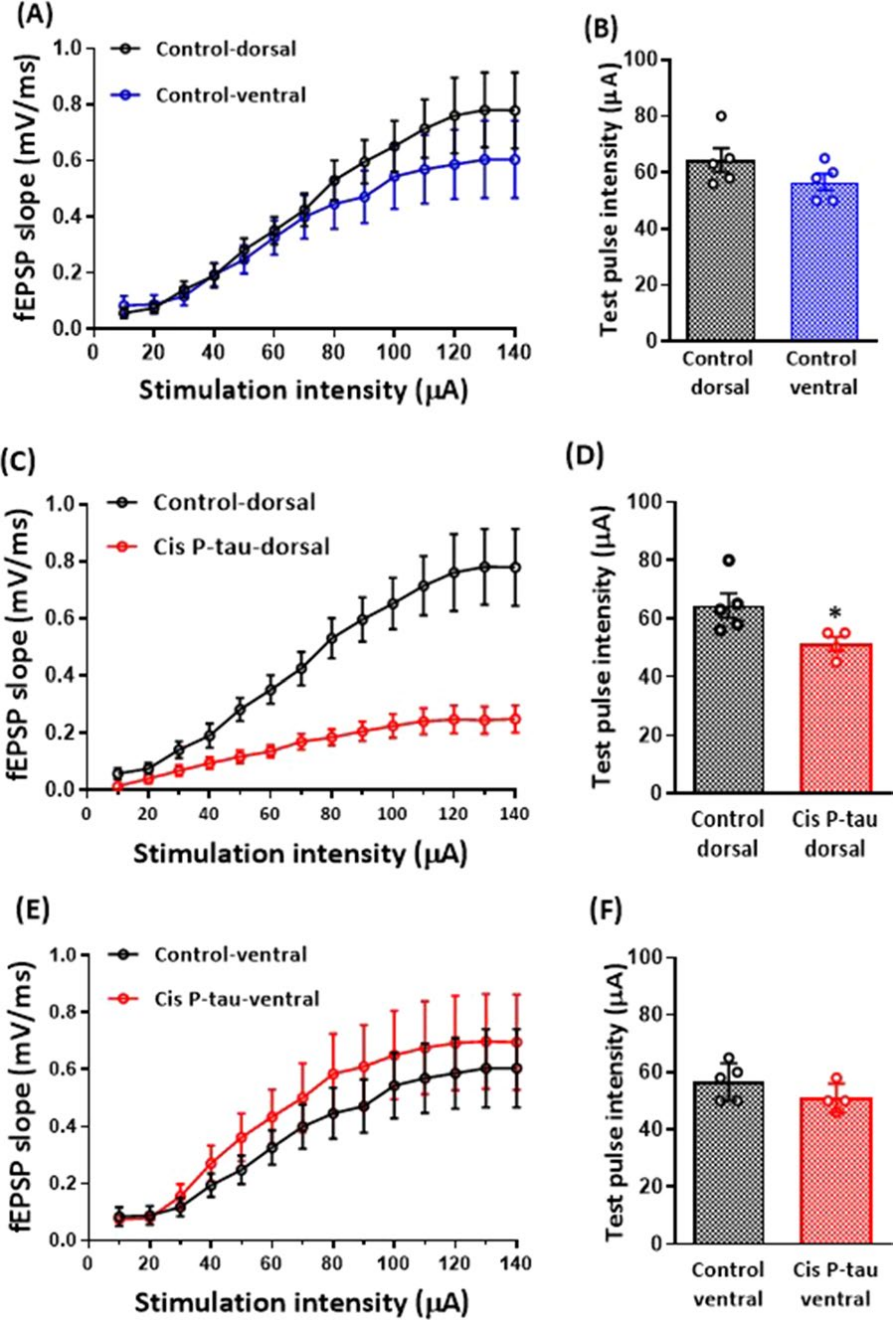
Basic synaptic transmission decreased in the dorsal hippocampus at 7 months after intra-hippocampal cis P-tau injection. Graphs show the basic synaptic transmission (I–O curve) **A** and test pulse intensity **B** in the dorsal vs. ventral hippocampal slices in the control group. Cis P-tau injection shifted the I-O curve to the right **C** and decreased the test pulse intensity **D** in the dorsal hippocampus in the cis P-tau compared to the control group. In the ventral hippocampal slices no significant difference observed in I-O curve **E** and test pulse intensity **F** between cis P-tau compared to the control group. All data represent mean ± SEM. * P < 0.05

We first compared the basal synaptic field potential responses in the dorsal and ventral CA1 stratum pyramidal in response to Schaffer collaterals stimulation in the control and cis P-tau groups. The fEPSP slope was measured in response to different stimulation intensities, and the I-O curves were constructed. Obtained results showed that the dorsal and ventral hippocampus had no significant differences in the I-O curve in the control group (Fig. 4A). There was no significant difference in test pulse intensity between the dorsal and ventral hippocampus in the control group (64.40 ± 4.23 and 56.60 ± 2.93 respectively, Fig. 4B). Compared to the control group, the I-O curve shifted to the right in the dorsal but not the ventral hippocampus in cis P-tau (Fig. 4C, E). Accordingly, the test pulse intensity significantly decreased in the dorsal hippocampus of the cis P-tau group compared to the control (51.25 ± 2,39 µA vs. 64.4 ± 4.23 µA; *P* < 0.05, Fig. 3D). No significant difference was observed in the test pulse intensity in the ventral hippocampus between cis P-tau (51.00 ± 2.93 µA) and control (51.00 ± 2.52 µA) groups (Fig. 4F).

### Cis P-tau disrupted LTP induction in the dorsal hippocampus

**Fig. 5:**
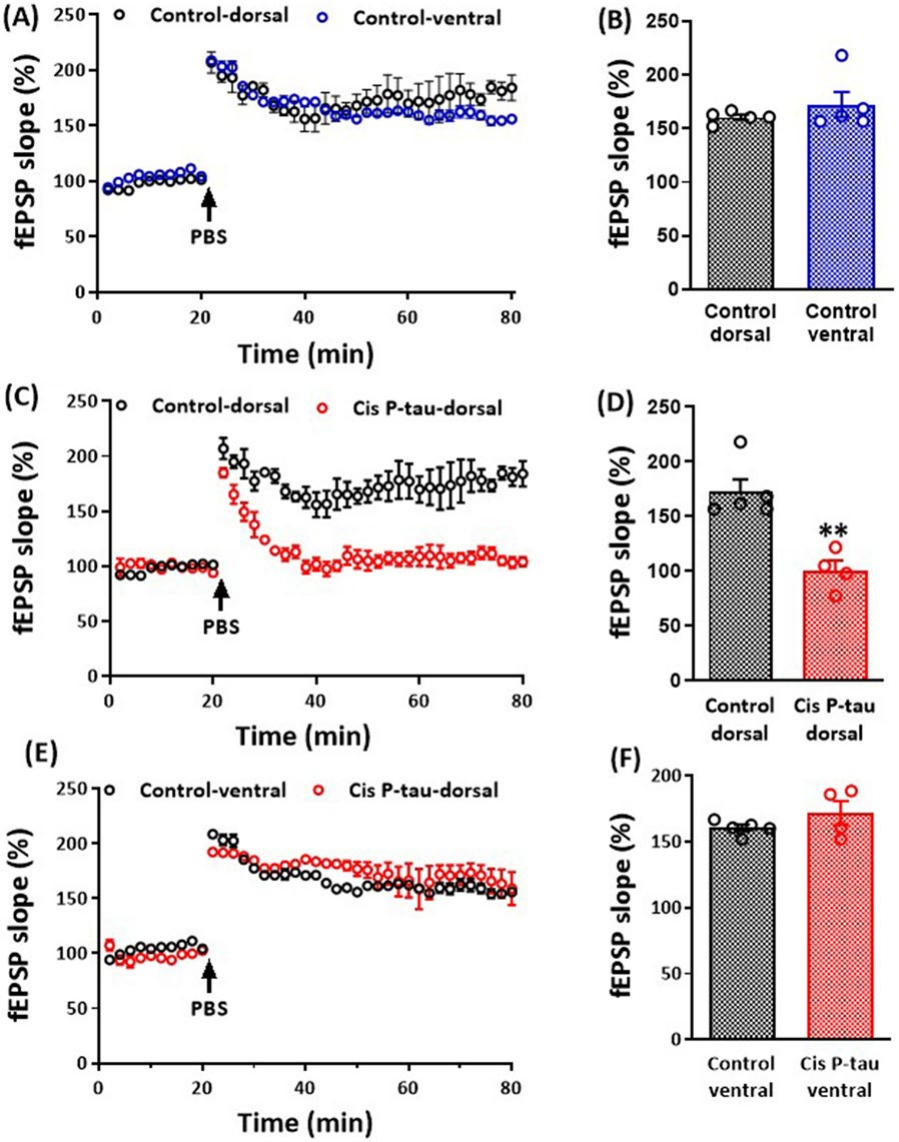
Cis P-tau injection disrupted LTP induction in the dorsal hippocampus at 7 months after injection. Graphs show the LTP magnitude in dorsal vs. ventral hippocampal slices in the control group (**A** and **B**). Cis P-tau injection decreased LTP magnitude in dorsal **C** and **D** but not in ventral **E** and **F** hippocampus in cis p-tau group compared to control group. All data represent mean ± SEM. ** P < 0.01 compared to the control group

In the next step, we assessed the generation of long-term potentiation (LTP) in fEPSP slope following primed burst stimulation (PBS) applying in the Schaffer collaterals in the stratum radiatum of the dorsal and ventral hippocampal CA1 area. There was not any significant difference between the dorsal (172.50 ± 11.66% of baseline) and ventral (160.60 ± 2.43% of baseline) hippocampal LTP magnitude in the control group (Fig. 5A, B).

Then, we compared the LTP magnitude in the dorsal and ventral hippocampus of the cis P-tau groups with the control group. Data demonstrated that intra-hippocampal cis P-tau injection significantly (*P* < 0.01) reduced the LTP magnitude in the dorsal hippocampus (106.10 ± 5.52% of baseline; Fig. 5C, D). However, there was no significant difference in LTP magnitude in the ventral hippocampus between the two groups (Fig. 5E, F).

### Cis P-tau decreased the number of survived cells in the hippocampus

**Fig. 6:**
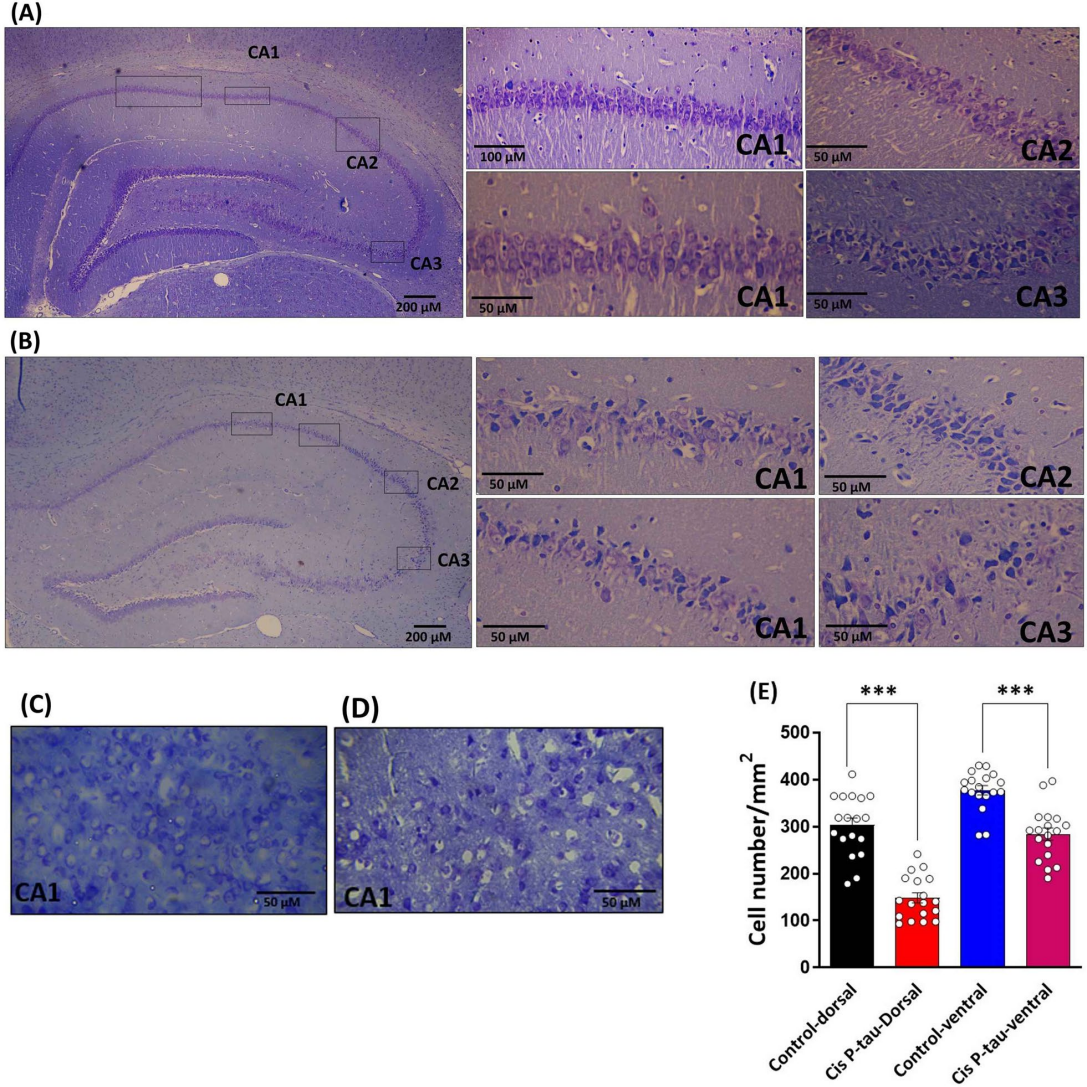
Cis P-tau decreased the hippocampal survived cells in the dorsal and ventral hippocampus at 7 months after injection. Representative hippocampal sections were stained with the Nissl method for evaluation of the survivor cells in control-dorsal **A** cis P-tau-dorsal **B** control-ventral **C** and cis P-tau-ventral **D** groups. Bar graph **E** shows the quantitative effect of cis P-tau injection on pyramidal survived cells in dorsal and ventral hippocampus compared to control group. All data represents mean ± SEM ^***^ P < 0.001

For assessing the changes in the number of survived cells in the dorsal and ventral hippocampus, Nissl staining was performed in cis P-tau and control groups. As Fig. 6 shows, the number of survived cells decreased significantly (*P* < 0.001) in the dorsal hippocampus of the cis-P tau group (148 ± 10.8) compared to the control (305 ± 15.2). Similarly, the number of survived cells decreased significantly (*P* < 0.001) in the ventral hippocampus of the cis-P tau group (284 ± 13.3) compared to the control group (377.4 ± 9.9). In addition, the reduction of survived cells in the dorsal hippocampus was higher than in the ventral hippocampus.

## Discussion

While it has been reported that bilateral intra-hippocampal injection of cis P-tau increases amyloid-beta accumulation and tau protein aggregation in the hippocampus at 2, 4, and 8 weeks after cis P-tau injection [15], results of the current study demonstrated that there was no intra-hippocampal β-amyloid and cis P-tau accumulation at 7 months after cis P-tau injection. However, a significant deficit in learning and memory and synaptic plasticity dysfunction was observed at 7 months after intra-hippocampal injection of cis P-tau.

A significant decrease in spontaneous alternation in the Y-maze test was observed following cis P-tau injection, which showed a deficit in working memory in the cis P-tau group compared to the control group at 7 months after injection. A normal working memory needs interplay between several areas of the brain, such as the ventral hippocampus and prefrontal cortex [19, 20]. Therefore, it may be postulated that following microinjection of cis P-tau into the dorsal hippocampus, an accumulation of cis-P tau in the ventral hippocampus and the prefrontal cortex probably caused neurodegeneration in both areas. In addition, animals in cis P-tau group spent less time in the center of Y-maze apparatus. It showed that when the subjects reached to the center point of the maze, they did not make a decision to go to next arm and moved without any goal. However, in the control group, after reaching the center point of the maze, the animals took more time to choose the correct arm. It has been reported that cis-P tau microinjection into the hippocampus may induce beta amyloid plaques and both cis P-tau and beta amyloid (Aβ1-42) induce each other and leads to similar and identical neurotoxicity [15]. Therefore, the results of the present study may be contributed to a tau-induced Alzheimer’s-like disease model in mice.

In this study, we used the Barnes maze, which is less stressful than the Morris water maze, especially for mice [21, 22]. In addition, many articles highlight the priority of mice (especially C57BL/6 J) to rats in Barnes maze studies due to their innate curiosity and tendency to escape into the small holes [23, 24].

Animals in cis P-tau group showed spatial learning and memory impairment in the Barnes maze test. The increase in primary and total errors and latency to the goal box and the decrease in using the direct strategy showed a significant impairment in learning processes in cis P-tau group. In addition, obtained data on probe test day showed that subjects in the cis P-tau group spent less time in the goal sector than the control, indicating memory impairment in this group. Similar cognition impairments were previously reported in Alzheimer’s like disease models in laboratory mice [25–27]. In line with the present data, Ramsden et al. in [28] reported that spatial memory was dramatically impaired in tauopathic mice at 7 and 9.5 months of age in the Morris water maze [28]. Brunden et al. [29] also showed an increase in the number of errors in the Barnes maze test in a transgenic mice model of tauopathy [29]. The spatial memory impairment of transgenic mice in Barnes and Morris water mazes may indicate hippocampal dysfunction.

The dorsal hippocampus has a crucial role in spatial learning and memory [30–32] while the ventral hippocampus is mainly involved in emotional procedures [33, 34]. Therefore, our data confirmed a significant dysfunction in dorsal hippocampus of Alzheimer’s like-disease animals. In consistent with our data, some studies reported the neuropathological changes in the hippocampus of transgenetic mice model of AD [35, 36]. In addition, O'Lear et al. reported that the APP/PS1 AD transgenic model mice spent less time in the correct zone than wild-type because of impaired spatial memory [37].

In the present study, distance and velocity had no significant differences between cis P-tau and the control groups, indicating no changes in motor activity between the two groups.

Since an AD brain dramatically loses synapses in the temporal areas, the changes in synaptic strength are important signs to show the magnitude of Alzheimer’-like disease models and tauopathogenesis in animal models [38]. According to the input–output curve, the basal synaptic function had no significant difference between the dorsal and ventral hippocampus of the control group. Consistent with our data, Tidball et al. [39], Milior et al. [40], and Schreurs et al. [41] showed that no difference was detected in the relationship between stimulus intensity and fEPSP slope in the dorsal versus the ventral hippocampus [39–41]. In contrast to the control group, in the cis P-tau group, excitability decreased in the dorsal region but had no change in the ventral. In line with our study, basic synaptic transmission was reduced in the hippocampus in a transgenic animal model of AD (5xFAD mice) [38]. In another study, Tulloch et al. [42] compared basic synaptic transmission in several Alzheimer's animal models and did not observe any significant difference between the control and the Alzheimer's groups. In line with our study, the basic synaptic transmission was slightly reduced (not significant) in mice AD-transgenic models of APP/PS1 + Tau [42].

LTP at Schaffer collateral synapses was not different in the dorsal and ventral hippocampus of the control group. This data is contrary to the study performed by Milior et al. [40]. They investigated the electrophysiological properties of CA1 pyramidal cells along the whole hippocampal dorsoventral axis in 2–3 months old mice. They found that LTP in Schaffer collateral synapses is lower in the ventral hippocampus than in the dorsal, a phenomenon that is related to more excitability of ventral pyramidal neurons compared to the dorsal region of the hippocampus [43, 44] Papaleonidopoulos et al. also reported that the dorsal hippocampus has a lower threshold for LTP induction compared to the ventral region in 28–38 days and 2–3 months old rats. Because the main difference between the present study and the previous report relates to the age of the subjects, we used 11-month-old mice. Therefore, it may be suggested that the difference in excitability of neurons between dorsal and ventral areas of the hippocampus disappears by aging. The lack of difference between hippocampal dorsal and ventral areas also confirms this hypothesis.

The present results also showed that hippocampal cis P-tau injection disrupted LTP induction in the dorsal hippocampus but did not significantly affect the ventral region. Similar to our data, Crouzin et al. reported that LTP was not induced in a mice model of Alzheimer's-like disease [38]. They concluded a reverse correlation between LTP incidence and pathophysiological changes in the hippocampus. Thus, the severity of Alzheimer's pathological changes is matched with the extent of the synaptic plasticity losses and, eventually, nerve damage occurrence. The decrease in the number of pyramidal cells and thickness of CA1 and CA3 regions have also been reported in Alzheimer’s-like disease induced by scopolamine [45] and in APP/PS1 transgenic mouse models of Alzheimer's disease [46]. In line with the previous studies, the present data also showed that the number of hippocampal neurons decreased after cis P-tau injection in dorsal and ventral areas. However, the percentage of decrease in the dorsal was higher than ventral area. This difference may be considered as a reason for LTP disruption in the dorsal, but not in the ventral hippocampus.

## Conclusion

The probable tau pathogenesis following intra-hippocampal cis P-tau resulted in working and spatial memory impairment and constructed a long-term Alzheimer's-like behavior for a long duration. In addition, basal synaptic transmission and synaptic plasticity were disrupted in the dorsal region of the cis P-tau group. The number of neurons decreased in the hippocampus, while this decrease was higher in the dorsal hippocampus than in the ventral. Of course, because cis P-tau was injected into the dorsal hippocampus and resulted in a higher neuronal loss (compared to the ventral area), it was logic to observe higher histological damage in the dorsal part, which was accompanied by impairment in synaptic potentiation and spatial learning and memory impairment. More research needs to assay the accumulation of beta-amyloid, cis P-tau, and apoptosis process in different hippocampal areas by immunohistochemistry.

## Methods

### Cis-P tau extraction

Pathogenic P-tau formation was induced by the traumatic brain injury (TBI) model [11]. In this model, TBI occurred by dropping a 450 g weight from 2 m height on skull of anesthetized adult male Wistar rats [47, 48]. It was purified from the brain extract after cis pT231- tau accumulation confirmation. The cortex tissues were separated and lysed in an extraction buffer (2 ml/g tissue). The contents of extraction solution include 20 mM PIPES pH 6.9, 1 mM MgSO4, 1 mM EGTA in the presence of 2 mM DTT, 1 mM PMSF, 1 mM EDTA, and 1 M NaCl. Then we pellet the homogenate by centrifugation at 6000 × g for 20 min at 4 ℃, and we sonicated the supernatant on ice four times (15 s on, the 30 s off) and boiled in 5 M NaCl for 10 min. We chilled the extract on ice and then ultra-centrifuged (Beckman coulter optima L-100XP) at 100,000 × g for 60 min at 4 ℃. The supernatant was dialyzed against PEM buffer (3 × 1). Finally, we purified tau protein by ion-exchange chromatography, as explained [49], and stored it at—80 ℃ until use.

### Animals

Thirty adult male C57BL/6 mice (4 months old, weighing 25–27 g) were obtained from Tarbiat Modares University (Tehran, Iran) and housed in 21 ± 2 ℃, 12 h light–dark cycle, with free access to food and water. Animals were divided into control and cis-P tau groups. Cis-P tau or its solvent (saline) were injected intra-hippocampally in cis-P tau and control groups respectively. All experiments were run 7 months after chemical injection.

### Stereotaxic surgery

Mice were anesthetized with an intraperitoneal injection of ketamine/xylazine (100 mg/kg to 10 mg/kg) and immobilized in a stereotaxic frame (Stoelting, USA). AD model was induced by cis-P tau injection (1 μg/1 μl) into the dorsal hippocampus (stereotaxic coordination: 2 mm posterior and 1.7 mm bilaterally to the right and left of bregma and—1.6 mm below dura [50]) in a volume of 2 μl over 6 min via a 10 μl Hamilton syringe using a microsyringe pump (WPI, UK). All microinjections were done at a speed of 0.5 μl/min, and the injection needle was left in place for an additional 10 min to allow the solution to diffuse from the tip entirely.

### Y- maze test

We selected a Y-shaped gray Plexiglas maze with 30 cm length, 10 cm width, and 15 cm height for the working memory task. The animal was put on the end of one arm to explore for 8 min session freely. An entry happens when all four mouse limbs are inside an arm. An alternation is determined as successive entries into all three arms. Next, after recording the number of arm entries and alternations, the percentage of the alternation behavior was calculated by the below formula:

$$Alternation \,precentage=\frac{Number \,of\, alternation}{Total \,number \,of \,arm \,entry}\times 100$$

A spontaneous alternation happens when a mouse enters a different arm of the maze in each of 3 consecutive arm entries (i.e., visit from A to B or C, which are designated to the other arms, respectively). In addition, incorrect trials are considered to travel back to a previously experienced arm, such as CBC moving. All movements were recorded using a computer-linked video camera mounted above the platform [51], and data were analyzed by Ethovision software 11 (Noldus Information Technology, Wageningen, The Netherlands).

### Barnes maze test

We assessed spatial hippocampal-dependent learning and memory using by Barnes maze test. The maze includes a circular platform (92 cm in diameter) with 20 holes (hole diameter: 5 cm) along with the surroundings. During the experiment, the mouse learned the spatial position of the goal box (17.5 cm in length, 7.5 cm in width, and 8 cm in height). Three-maze cues were placed all around the room to show the location of the goal box hole. In the pre-training trial, the mouse was put in the maze's center in a white-colored cubed start box (12.5 cm × 8 cm). After 10 s, the start box was raised, and the mouse learned to enter the goal box by guiding it to the goal box and staying there for 2 min. After the pre-training trial, the first trial began. At the onset of each trial, the mouse was put in the start box, and 10 s later, a light was turned on, the box was raised, and the mouse explored the maze freely. The trial finished when the mouse entered the goal box or after 5 min had elapsed. After entering the mouse into the goal box, the light was turned off, and the mouse remained in the goal box for 1 min.

Mice trained for four trials (at 15 min intervals) per day for 4 days. After each trial, the maze was cleaned with 70% ethylic alcohol solution. A probe trial was done on the 5 day when the goal box was closed to assess maze learning and memory retention. The probe experiment allows determining whether trained animals use environmental cues to create a spatial map of their environment and find a hole that was previously a goal box. The delay and time spent to find the last correct hole was measured. Total trials were recorded by using a ceiling-mounted video camera.

The measured behavioral parameters included: 1—Primary and total latency evaluated as the time spent by the mouse to find the goal box for the first time (primary latency) and entering (total latency) during a learning trial; 2—Errors measured as the number of incorrect holes explore before finding (primary errors) and entering (total errors) the goal box. Errors are explained as exploring any hole that does not contain the goal box; 3—Total distance and velocity for each trial are also calculated by using EthoVision XT; 4—For each trial, the search strategy (exploration patterns) is classified as direct (moving directly to the target hole), serial (systematic search of sequential holes in a clockwise or counterclockwise direction), and random (unordered and random exploration of the maze); 5—The frequency of target hole exploration was assessed by the goal sector (GS) parameter, and it is the sum target and a neighbor right or left holes explorations divided by 3; 6—The frequency of non-target hole exploration was assessed by the non-goal sector (NGS): the sum of explorations of the 17 non-goal holes divided by 17; 7—Goal sector preference: the ratio of GS to NGS explorations; 8—Target-seeking activity: the total explorations for whole, divided by 20 [24].

### Field potential recording in the hippocampal slices

The mice were anesthetized with carbon dioxide (CO_2_) and decapitated at 7 months after bilateral hippocampal cis-P tau injection. We removed the mouse brain rapidly, and then the fresh brain was transferred into a chilled artificial cerebrospinal fluid (aCSF) chamber. This chamber bubbled with carbogen (95% O_2_ and 5% CO_2_). The aCSF contained (in mµ): NaCl 124, NaHCO_3_ 26, KH_2_PO_4_ 1.25, KCl 5, CaCl_2_ 2, MgCl_2_ 2.06, and d-glucose 10 and its pH was 7.3–7.4. Next, we prepared coronal 400 µm thick slices containing the hippocampus using a vibratome (model VT 1200, Leica, Germany). After slice preparation, we put the slices in a recovery chamber for at least 60 min at room temperature. Then we transferred slices (one by one) to an interface-type recording chamber containing 32 ℃ aCSF solution in a warm, humid oxygenated environment.

We recorded field potentials from the stratum radiatum in the dorsal and ventral hippocampus. We used a stimulating electrode (stainless steel, Teflon coated, A–M Systems, USA) that was placed on the Schaffer collateral path and a recording glass electrode (borosilicate, O.D.: 1.5 mm, I.D.: 0.86 mm, Sutter instrument, USA). The recording electrode (2–5 MΩ) was filled with aCSF and was placed on the stratum radiatum of hippocampal CA1. A reference electrode was also put in the recording chamber. The recording electrode transferred signals to an amplifier (ME208300, Nihon-kohden, Japan), and the signals were visualized by custom-made software (Potentialize; ScienceBeamCo., Iran). We plotted the Input/output curve to calculate test pulse intensity. The evoked field potential was recorded from the CA1 area at the test pulse intensity (50% of an intensity producing maximum response) for 20 min. Then, primed-burst stimulation (PBS; a single pulse followed 170 µs later by a burst of 10 pulses at 200 Hz, and the entire train was repeated ten times) was applied, and post-PBS responses were recorded for 60 min.

### Tissue processing and sectioning

The mice were deeply anesthetized with ketamine/xylazine (100 mg/kg to 10 mg/kg) 7 months after cis P-tau injection. Then transcardial perfusion was performed with phosphate-buffered saline (20 mL) followed by 4% phosphate-buffered formalin (15–20 mL). Brain tissues were fixed overnight in the solution (4% paraformaldehyde in phosphate-buffered saline), next embedded in OCT compound (Sakura; Finetek; Torrance, CA), and cut into 8 μm thick serial sections.

### Nissl staining

The number of survived cells was determined using the Nissl staining method. In brief, the sections were rehydrated with graded series alcohols (96%, 80%, and 70%) and stained with 0.1% Cresyl Fast Violet (Merck, Germany) at room temperature for 2 min. After washing, the sections were dehydrated by a graded series of alcohols (70%, 80%, 96%, and 100%). Then they were cleaned in xylene, cover slipped with Entellan (Merck, Chemical, Germany), and photographed. An Olympus BX-51 microscope and DP72 camera captured consecutive images at 400 × magnification. Using a grid (200 μm × 200 μm), the images were randomly assigned, and six squares were counted to measure survived cells, which were calculated as the number of cells/mm^2^.

### Immunofluorescence

After washing with PBS-Tween, brain sections were permeabilized for 10 min with 0.2% (v/v) Triton X-100 and blocked for 1 h with NGS 10%. Afterward, the samples were incubated with the following primary antibodies: cis pT231-tau mAb (1:500, gift from KP. Lu), and Aβ oligomers (1:500, Abcam) at 4 ℃ in a moist and humid chamber overnight. A secondary antibody anti-rabbit or anti-mouse was added after washing the samples at 37 ℃ for one hour (Alexa Fluor 488, Thermo Fisher Scientific, Rockford, USA). DAPI was used for staining the nuclei. The samples were visualized by a fluorescent microscope (Olympus, BX51 with Olympus DP72 digital camera), and the images were analyzed by using ImageJ software v1.43 (NIH, Bethesda, MD, USA).

### Statistical analysis

Spontaneous alternation, percent time in center point, total distance, test pulse, and percentage of potentiation were analyzed by unpaired t-test. Primary and total errors and latency, as well as field potentials (before and after LTP induction), were analyzed using two-way ANOVA followed by Sidak's multiple comparisons test. The correlation between the direct strategies in trial days was analyzed using the correlation test and Pearson correlation coefficient. The values were expressed as means ± standard error of the mean (SEM). All statistical analyses were conducted using Graphpad Prism (version 6.0). The probability level is interpreted as statistically significant when *P* < 0.05.

## Data Availability

The data that support the findings of this study are available on request from the corresponding author.

## References

[1] McKeith I, Cummings J (2005). Behavioural changes and psychological symptoms in dementia disorders. Lancet Neurol.

[2] McKhann G, Drachman D, Folstein M, Katzman R, Price D, Stadlan EM (1984). Clinical diagnosis of Alzheimer's disease: report of the NINCDS-ADRDA work group under the auspices of department of health and human services task force on Alzheimer's disease. Neurology.

[3] Goedert M, Spillantini MG (2006). A century of Alzheimer's disease. Science.

[4] Nakamura K, Greenwood A, Binder L, Bigio EH, Denial S, Nicholson L (2012). Proline isomer-specific antibodies reveal the early pathogenic tau conformation in Alzheimer's disease. Cell.

[5] Hyman BT, Augustinack JC, Ingelsson M (2005). Transcriptional and conformational changes of the tau molecule in Alzheimer's disease. Biochem Biophys Acta.

[6] Lee VM, Goedert M, Trojanowski JQ (2001). Neurodegenerative tauopathies. Annu Rev Neurosci.

[7] Moreno-Gonzalez I, Soto C (2011). Misfolded protein aggregates: mechanisms, structures and potential for disease transmission. Semin Cell Dev Biol.

[8] Soto C (2003). Unfolding the role of protein misfolding in neurodegenerative diseases. Nat Rev Neurosci.

[9] Ballatore C, Lee VM, Trojanowski JQ (2007). Tau-mediated neurodegeneration in Alzheimer's disease and related disorders. Nat Rev Neurosci.

[10] Mandelkow EM, Mandelkow E (2012). Biochemistry and cell biology of tau protein in neurofibrillary degeneration. Cold Spring Harb Perspect Med.

[11] Kondo A, Shahpasand K, Mannix R, Qiu J, Moncaster J, Chen C-H (2015). Antibody against early driver of neurodegeneration cis P-tau blocks brain injury and tauopathy. Nature.

[12] Bibow S, Ozenne V, Biernat J, Blackledge M, Mandelkow E, Zweckstetter M (2011). Structural impact of proline-directed pseudophosphorylation at AT8, AT100, and PHF1 epitopes on 441-residue tau. J Am Chem Soc.

[13] Steinhilb ML, Dias-Santagata D, Fulga TA, Felch DL, Feany MB (2007). Tau phosphorylation sites work in concert to promote neurotoxicity in vivo. Mol Biol Cell.

[14] Smith DH, Uryu K, Saatman KE, Trojanowski JQ, McIntosh TK (2003). Protein accumulation in traumatic brain injury. NeuroMol Med.

[15] Pourhamzeh M, Joghataei MT, Mehrabi S, Ahadi R, Hojjati SMM, Fazli N (2021). The interplay of tau protein and β-amyloid: while tauopathy spreads more profoundly than amyloidopathy, both processes are almost equally pathogenic. Cell Mol Neurobiol.

[16] Zhang Y, Wu F, Iqbal K, Gong C-X, Hu W, Liu F (2019). Subacute to chronic Alzheimer-like alterations after controlled cortical impact in human tau transgenic mice. Sci Rep.

[17] Yoshiyama Y, Higuchi M, Zhang B, Huang SM, Iwata N, Saido TC (2007). Synapse loss and microglial activation precede tangles in a P301S tauopathy mouse model. Neuron.

[18] Nelson PT, Alafuzoff I, Bigio EH, Bouras C, Braak H, Cairns NJ (2012). Correlation of Alzheimer disease neuropathologic changes with cognitive status: a review of the literature. J Neuropathol Exp Neurol.

[19] Swonger AK, Rech RH (1972). Serotonergic and cholinergic involvement in habituation of activity and spontaneous alternation of rats in a Y maze. J Comp Physiol Psychol.

[20] Sarnyai Z, Sibille EL, Pavlides C, Fenster RJ, McEwen BS, Toth M (2000). Impaired hippocampal-dependent learning and functional abnormalities in the hippocampus in mice lacking serotonin(1A) receptors. Proc Natl Acad Sci USA.

[21] Harrison FE, Hosseini AH, McDonald MP (2009). Endogenous anxiety and stress responses in water maze and Barnes maze spatial memory tasks. Behav Brain Res.

[22] Holmes A, Wrenn CC, Harris AP, Thayer KE, Crawley JN (2002). Behavioral profiles of inbred strains on novel olfactory, spatial and emotional tests for reference memory in mice. Genes Brain Behav.

[23] Bach ME, Hawkins RD, Osman M, Kandel ER, Mayford M (1995). Impairment of spatial but not contextual memory in CaMKII mutant mice with a selective loss of hippocampal LTP in the range of the theta frequency. Cell.

[24] Gawel K, Gibula E, Marszalek-Grabska M, Filarowska J, Kotlinska JH (2019). Assessment of spatial learning and memory in the Barnes maze task in rodents-methodological consideration. Naunyn Schmiedeberg Arch Pharmacol.

[25] King DL, Arendash GWJBR (2002). Maintained synaptophysin immunoreactivity in Tg2576 transgenic mice during aging correlations with cognitive impairment. Brain Res.

[26] Koopmans G, Blokland A, van Nieuwenhuijzen P, Prickaerts J (2003). Assessment of spatial learning abilities of mice in a new circular maze. Physiol Behav.

[27] Moffat SD, Resnick SM (2002). Effects of age on virtual environment place navigation and allocentric cognitive mapping. Behav Neurosci.

[28] Ramsden M, Kotilinek L, Forster C, Paulson J, McGowan E, SantaCruz K (2005). Age-dependent neurofibrillary tangle formation, neuron loss, and memory impairment in a mouse model of human tauopathy (P301L). J Neurosci Off J Soc Neurosci.

[29] Brunden KR, Zhang B, Carroll J, Yao Y, Potuzak JS, Hogan AM (2010). Epothilone D improves microtubule density, axonal integrity, and cognition in a transgenic mouse model of tauopathy. J Neurosci Off J Soc Neurosci.

[30] Moser E, Moser MB, Andersen P (1993). Spatial learning impairment parallels the magnitude of dorsal hippocampal lesions, but is hardly present following ventral lesions. Journal Neurosci Off J Soc Neurosci.

[31] Fanselow MS, Dong H-W (2010). Are the dorsal and ventral hippocampus functionally distinct structures?. Neuron.

[32] Bannerman DM, Sprengel R, Sanderson DJ, McHugh SB, Rawlins JNP, Monyer H (2014). Hippocampal synaptic plasticity, spatial memory and anxiety. Nat Rev Neurosci.

[33] Bannerman DM, Rawlins JN, McHugh SB, Deacon RM, Yee BK, Bast T (2004). Regional dissociations within the hippocampus–memory and anxiety. Neurosci Biobehav Rev.

[34] Bannerman DM, Grubb M, Deacon RM, Yee BK, Feldon J, Rawlins JN (2003). Ventral hippocampal lesions affect anxiety but not spatial learning. Behav Brain Res.

[35] Frautschy SA, Yang F, Irrizarry M, Hyman B, Saido TC, Hsiao K (1998). Microglial response to amyloid plaques in APPsw transgenic mice. Am J Pathol.

[36] Irizarry MC, Soriano F, McNamara M, Page KJ, Schenk D, Games D (1997). Abeta deposition is associated with neuropil changes, but not with overt neuronal loss in the human amyloid precursor protein V717F (PDAPP) transgenic mouse. J Neurosci Off J Soc Neurosci.

[37] O'Leary TP, Brown RE (2009). Visuo-spatial learning and memory deficits on the Barnes maze in the 16-month-old APPswe/PS1dE9 mouse model of Alzheimer's disease. Behav Brain Res.

[38] Crouzin N, Baranger K, Cavalier M, Marchalant Y, Cohen-Solal C, Roman FS (2013). Area-specific alterations of synaptic plasticity in the 5XFAD mouse model of Alzheimer's disease: dissociation between somatosensory cortex and hippocampus. PLoS ONE.

[39] Tidball P, Burn HV, Teh KL, Volianskis A, Collingridge GL, Fitzjohn SM (2017). Differential ability of the dorsal and ventral rat hippocampus to exhibit group I metabotropic glutamate receptor-dependent synaptic and intrinsic plasticity. Brain Neurosci Adv.

[40] Milior G, Di Castro MA, Sciarria LP, Garofalo S, Branchi I, Ragozzino D (2016). Electrophysiological properties of CA1 pyramidal neurons along the longitudinal axis of the mouse hippocampus. Sci Rep.

[41] Schreurs A, Sabanov V, Balschun D (2017). Distinct properties of long-term potentiation in the dentate gyrus along the dorsoventral axis: influence of age and inhibition. Sci Rep.

[42] Tulloch J, Netsyk O, Pickett EK, Herrmann AG, Jain P, Stevenson AJ (2021). Maintained memory and long-term potentiation in a mouse model of Alzheimer's disease with both amyloid pathology and human tau. Eur J Neurosci.

[43] Papaleonidopoulos V, Trompoukis G, Koutsoumpa A, Papatheodoropoulos C (2017). A gradient of frequency-dependent synaptic properties along the longitudinal hippocampal axis. BMC Neurosci.

[44] Dobrunz LE, Stevens CF (1997). Heterogeneity of release probability, facilitation, and depletion at central synapses. Neuron.

[45] Mandour DA, Bendary MA, Alsemeh AE (2021). Histological and imunohistochemical alterations of hippocampus and prefrontal cortex in a rat model of Alzheimer like-disease with a preferential role of the flavonoid "hesperidin". J Mol Histol.

[46] Lin R, Li L, Zhang Y, Huang S, Chen S, Shi J (2018). Electroacupuncture ameliorate learning and memory by improving N-acetylaspartate and glutamate metabolism in APP/PS1 mice. Biol Res.

[47] Amorini AM, Lazzarino G, Di Pietro V, Signoretti S, Lazzarino G, Belli A (2016). Metabolic, enzymatic and gene involvement in cerebral glucose dysmetabolism after traumatic brain injury. Biochem Biophys Acta.

[48] Di Pietro V, Lazzarino G, Amorini AM, Tavazzi B, D'Urso S, Longo S (2014). Neuroglobin expression and oxidant/antioxidant balance after graded traumatic brain injury in the rat. Free Radical Biol Med.

[49] Shahpasand K, Uemura I, Saito T, Asano T, Hata K, Shibata K (2012). Regulation of mitochondrial transport and inter-microtubule spacing by tau phosphorylation at the sites hyperphosphorylated in Alzheimer's disease. J Neurosci Off J Soc Neurosci.

[50] Franklin KBJ, Paxinos G. The Mouse Brain in Stereotaxic Coordinates: Academic Press; 1997.

[51] Kraeuter AK, Guest PC, Sarnyai Z (2019). The Y-Maze for Assessment of Spatial Working and Reference Memory in Mice. Methods Mol Biol (Clifton, NJ).

